# Clinical, hematological, and biochemical studies on hypozincemia in neonatal calves in Egypt

**DOI:** 10.14202/vetworld.2021.314-318

**Published:** 2021-02-03

**Authors:** Mamdouh M. El-Maghraby, Ahmed E. Mahmoud

**Affiliations:** Department of Veterinary Medicine, Faculty of Veterinary Medicine, Suez Canal University, Ismailia 41522, Egypt

**Keywords:** alopecia, calf, complete blood count, skin lesions, zinc deficiency

## Abstract

**Background and Aim:**

Zinc has a wide spectrum of biological activities and its deficiency has been related to various dysfunctions. This study aimed to clarify the clinical, hematological, and biochemical changes in Holstein dairy calves with naturally occurring hypozincemia before and after treatment.

**Materials and Methods:**

This study was carried out on 25 Holstein dairy calves <1 month of age in the El-Salhya Dairy Farm, Al-Sharqiya Province, Egypt. Calves were born from apparent healthy dams without any clinical signs of zinc deficiency. They were divided into two groups. The first group (G1) included five clinically healthy calves that were used as controls. The second group (G2) included 20 calves suffering from alopecia and skin lesions. The diseased calves were then treated by oral administration of zinc oxide at the rate of 80 mg/day for 10 successive days and then 20 mg/week for 2 weeks (G3). A total of 90 samples, whole blood and serum samples were collected during the study across all groups. Whole blood was evaluated for complete blood count and serum was used to estimate total protein, albumin, globulin, zinc, calcium, magnesium, phosphorus, and the activity of alkaline phosphatase (ALP) and aspartate aminotransferase.

**Results:**

The diseased calves had macrocytic normochromic anemia. Total leukocytes, neutrophils, and lymphocytes were significantly reduced in the diseased calves than in the control and treated groups. Biochemical analysis of serum revealed a highly significant decrease in the globulin, zinc, and calcium concentrations in the diseased calves than in the control and treated groups. ALP activity was significantly lower in the diseased and treated calves than in control. There were no differences in any other parameters between the groups.

**Conclusion:**

Zinc deficiency naturally occurring in calves caused clinical, hematological, and biochemical alterations such as alopecia, skin abnormalities, and macrocytic normochromic anemia. In addition, zinc deficiency altered the cell-mediated immunity as indicated by leukopenia and lymphopenia. These alterations were improved by oral administration of zinc oxide.

## Introduction

Trace elements are fundamental for animal health, growth, production, reproduction, and immunity [1]. Zinc is one of the most important trace elements, with a wide spectrum of biological activities. It is an integral component of more than 300 metalloenzymes and is vital for DNA stabilization and gene expression [2,3]. In addition, it plays a key role in immunological responses, and zinc deficiency adversely affects the cell-mediated immune system [4,5]. Zinc is also essential for the biosynthesis of fatty acids and Vitamin A metabolism [6] and is considered a vital element for the activity of several hormones, such as glucagon, insulin, growth hormone, and sex hormones [7]. Zinc is particularly important in rapidly dividing cells, including those of the epidermis [8]. Its deficiency has been linked to various dysfunctions and alterations of normal cell metabolism, which leads to growth rate retardation [9]. Dietary zinc supplementation is essential for increased growth and immune competence of livestock [10]. Zinc sulfate and oxide are the major forms of zinc salts added to diets of calves [11,12]. Moreover, zinc sulfate can be added to calves’ every milk meal for 14 days [13].

Whereas, the previous studies have outlined experimental zinc deficiency in sheep [4] and natural cases of zinc deficiency in Iraqi buffalo calves and cattle ([9,14], respectively) without treatment trials. Therefore, this study was designed to elucidate the clinical, hematological, and biochemical alterations of naturally occurring hypozincemia in Holstein dairy calves from apparently healthy cows in Egyptian fields. The study further aimed to trial a treatment for the deficient calves and to monitor its efficacy.

## Materials and Methods

### Ethical approval

All procedures used in the present study were approved by the Scientific Research Ethics Committee on animal researches, Faculty of Veterinary Medicine, Suez Canal University, Egypt.

### Study period and location

The present study was carried out from July to August 2018, in Alsharqaia Province, Egypt.

### Animal and study design

This study included 25 Holstein dairy calves (12 males and 13 females) < 1-month-old with body weights of 60-65 kg. Calves were born from apparent healthy dams with no clinical signs of zinc deficiency. Calves were reared in El-Salhya Dairy Farm, Elsharqaia Governorate, Egypt, from July to August 2018. There were two groups: 5 healthy control calves (G1) and 20 calves suffering from alopecia and skin lesions (G2). The diseased calves were then treated with orally administered zinc oxide at the rate of 80 mg/day for 10 successive days and then 20 mg/week for 2 weeks [11] and analyzed as a third group (G3). All calves were housed in a group hutch on an open yard. Calves were fed three meals of milk at a rate of 2-3 L/meal/calf and calf starter according to the Association Official Analytical Chemists International [15] (Table-1), and tap water was also available *ad libitum*. Clinical examinations were carried out on all animals according to Constable *et al*. [16], and all animals were free from internal and external parasites.

**Table-1:** Ingredient and chemical composition of calf starter ration.

Ingredients	Percent
Ingredient (%, as fed basis)	
Maize grain	52.5
Corn gluten feed	17
Soybean meal 44% protein	28
Limestone powder	1
Iodized sodium chloride	0.5
Trace mineral and vitamin premix*	1
Total	100
Chemical composition (on dry matter basis)	
Dry matter (%)	88.43
Crude protein (%)	18.2
Ether extract (%)	3.41
Crude fiber (%)	3.87
Ash (%)	2.66
Metabolizable energy, Mcal/kg	3.04
Protein-energy ratio, g/Mcal	59.86
Ca:P ratio	2:1

* Each kilogram of trace mineral and vitamin premix contains: Vitamin A (50,000 IU); Vitamin D3 (10,000 IU); Vitamin E (0.1 g); calcium (196 g); phosphorus (96 g); sodium (71 g); magnesium sulfate (19 g); iron sulfate (3 g); copper sulfate (0.3 g); manganese (2 g); zinc sulfate (3 g); cobalt sulfate (0.1 g); potassium iodide (0.1 g); sodium selenate (0.001 g)

### Sampling

A total of 90 blood samples were collected as follows: 10 samples from G1, 40 from G2 (before treatment), and 40 from G3 (after the end of the treatment course). Blood samples were collected from the jugular vein of each calf into tubes containing ethylenediaminetetraacetic acid (for a complete blood count) or a plain tube in which it was allowed to clot for 30-60 min and then centrifuged at 3000 rpm for 20 min to separate sera. Sera samples were transferred into microcentrifuge tubes and then stored at −20°C for later biochemical analysis [17].

### Hematological analysis

Total erythrocyte count (red blood cells [RBCs]), hemoglobin (Hb) concentration, packed cell volume (PCV%), mean corpuscular volume (MCV), mean corpuscular hemoglobin (MCH), MCH concentration (MCHC), total leukocyte count (TLC), differential leukocyte count, and platelet count (PLT) were estimated according to the methods adopted by Feldman *et al*. [18].

### Biochemical analysis

Total protein (TP), albumin (ALB), zinc, calcium, magnesium, and phosphorus concentrations were estimated calorimetrically using commercial kits supplied by Spectrum, Egypt. Serum enzymatic activities of alkaline phosphatase (ALP) and aspartate aminotransferase (AST) were estimated calorimetrically using commercial kits supplied by Elitech (Egypt). Globulin was calculated as the difference between TP and ALB.

### Statistical analysis

The data were statistically analyzed using one-way analysis of variance with SPSS version 20.0 (IBM Corp., NY, USA). [19]. The differences were considered significant if p≤0.05.

## Results

**Figure-1:**
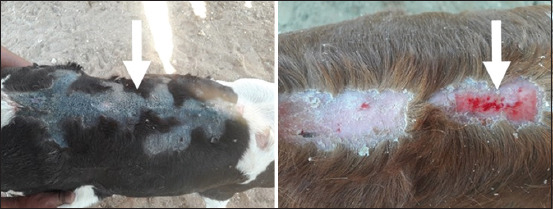
Cutaneous lesions (arrows) on the back of zinc-deficient calf. The lesions show alopecia and skin lesions including rough, thickened, and wrinkled skin erythematous lesions after removal of crust and pale mucus membrane.

**Figure-2:**
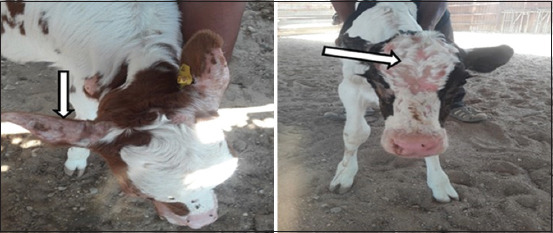
Cutaneous lesions (arrows) on the external pinna and forehead of zinc-deficient calf manifested by alopecia.

Alopecia was seen in 20 out of the 25 calves (80%). The alopecic lesions were mostly seen in the back, external pinna, forehead, periocular, shoulder, and lateral aspect of the hindlimbs (Table-2, Figures-1 and 2). The abnormal skin included rough, thickened, and wrinkled skin erythematous lesions after removal of crusts. Body temperature, respiratory rate, and heart rate were not significantly different between groups. The mucous membrane appeared pale in diseased calves than a bright red color in healthy and recovered calves (Table-3). The diseased calves improved 30 days after the onset of treatment with zinc oxide at the previously prescribed dose.

**Table-2:** Distribution of skin lesions in different area of animal body in diseased calves.

Animal body	Number of calves (n=20)	Percent
Back	8	40
External pinna	5	25
Forehead	3	15
Periocular	2	10
Shoulder	1	5
Lateral aspect of hindlimbs	1	5

**Table-3:** Clinical parameters in neonatal calves suffered from hypozincemia.

Groups	Parameters			

	Body temperature (°C)	Respiratory rate (per min)	Heart rate (per min)	Mucous membrane
Control (G1)	39.12^A^±0.67	25.80^A^±3.11	82.32^A^±2.40	Bright red
Diseased calves (G2)	38.72^A^±0.45	28.47^A^±1.23	77.54^A^±5.11	Pale
Treated calves (G3)	38.53^A^±0.20	26.72^A^±1.88	80.44^A^±3.70	Bright red

Means carrying the same superscripts in the same column are non-significantly different at p<0.05

Table-4 shows that the mean Hb concentration, PCV%, and RBC count were significantly lower (p<0.01) in the diseased calves than in the control group. MCV was significantly higher (p<0.01) in the diseased calves than in the control groups. MCHC and MCH showed no significant differences between any groups. Macrocytic normochromic anemia was seen in zinc-deficient calves.

**Table-4:** Mean values±standard error of hematological parameters in neonatal calves suffered from hypozincemia.

Parameters	Control (G1)	Diseased calves (G2)	Treated calves (G3)
Hemoglobin (g/dL)	12.32^A^±0.40	8.95^B^±0.30	11.95^A^±0.29
PCV%	38.2^A^±0.35	27.24^B^±0.80	35.94^A^±0.75
RBCs (×10^6^/µL)	6.12^A^±0.14	3.16^B^±0.12	5.78^A^±0.17
MCV (fL)	62.64^B^±1.93	87.04^A^±2.49	62.89^B^±2.30
MCH (pg)	20.18^a^±0.75	21.19^a^±0.45	20.92^a^±0.82
MCHC (g/dL)	32.24^a^±0.98	31.66^a^±0.46	33.37^a^±0.85
TLC (×10^3^/µL)	8.66^A^±1.03	5.66^B^±0.52	7.95^A^±0.45
Neutrophils (10^3^/µL)	4.34^A^±0.58	2.85^B^±0.25	4.00^A^±0.26
Lymphocytes (10^3^/µL)	3.56^A^±0.41	2.20^B^±0.22	3.32^A^±0.19
Monocytes (10^3^/µl)	0.47^a^±0.07	0.44^ab^±0.04	0.42^ab^±0.03
Eosinophils (10^3^/µL)	0.30^a^±0.05	0.21^a^±0.02	0.25^a^±0.01
Basophils (10^3^/µL)	0.00±0.0	0.00±0.0	0.00±0.0
Blood platelets (×10^3^/µL)	188.00^a^±6.81	176.73^a^±15.06	206.0^a^±5.21

Means carrying different small and capital superscripts in the same column are significantly different at p≤0.05 and p≤0.01, respectively. Means carrying the same superscripts in the same column are non-significantly different at p<0.05. PCV=Packed cell volume, RBC=Red blood cell, MCV=Mean corpuscular volume, MCH=Mean corpuscular hemoglobin, MCHC=Mean corpuscular hemoglobin concentration, TLC=Total leukocyte count

TLC, neutrophils, and lymphocytes were significantly reduced (p<0.01) in the diseased calves than in the control and treated groups. Monocytes, eosinophils, and PLT were not significantly different among any group.

Table-5 shows that the serum levels of globulin, zinc, and calcium were significantly lower (p<0.01) in the hypozincemic calves than in the control and treated groups. ALP activity was significantly lower (p<0.01) in both the diseased and treated calves than in the control group. Serum concentrations of TP, ALB, magnesium, and phosphorus and AST activity did not differ among the groups.

**Table-5:** Mean values±standard error of level of some biochemical parameters in neonatal calves suffered from hypozincemia.

Parameters	Control (G1)	Diseased calves (G2)	Treated calves (G3)
Total protein (g/dL)	8.16^a^±0.71	7.74^a^±0.21	7.82^a^±0.14
Albumin (g/dL)	5.80^a^±0.36	5.92^a^±0.23	5.67^a^±0.19
Globulins (g/dL)	2.36^a^±0.54	1.53^b^±0.15	2.15^a^±0.15
Zinc	27.60^a^±1.43	13.93^b^±0.43	25.20^a^±1.12
Calcium	11.53^a^±0.93	8.96^b^±0.24	11.91^a^±0.40
Magnesium	1.77^a^±0.15	1.68^a^±0.11	1.99^a^±0.11
Phosphorous	3.55^a^±0.43	3.67^a^±0.18	4.19^a^±0.15
AST (U/L)	119.77^a^±3.02	124.58^a^±2.82	121.91^a^±2.09
ALP (U/L)	81.26^a^±1.19	66.16^b^±2.18	64.52^b^±2.37

Means carrying different superscripts in the same column are highly significantly different at p≤0.01. Means carrying the same superscripts in the same column are non-significantly different at p<0.05. AST=Aspartate aminotransferase, ALP=Alkaline phosphatase

## Discussion

In the animals included in our study, alopecia on the back, external pinna, forehead, periocular, shoulder, and lateral aspect of the hindlimbs represented the most prominent gross lesions in the hypozincemic calves, similar to the lesions recorded by Sharma and Joshi [20] and Al-Saad *et al*. [21] in calves and Ibrahim *et al*. [4] in sheep. The parakeratosis, thickening, hardening, and cracking of the skin we observed are common signs of zinc deprivation in all animal species [16]. The concentration of zinc is usually higher in the epidermis than in the dermis, as zinc is required for the active proliferation and differentiation of epidermal keratinocytes [22], which may explain the dermatological changes in deficient calves as observed in this study.

The macrocytic normochromic anemia observed in our diseased calves was similar to the results recorded by Alsaad *et al*. [9] and Al-Saad *et al*. [21] in cattle and sheep, respectively. Zinc deficiency can lead to impairment of cell replication and protein synthesis and, therefore impaired generation of blood cells [23], which may explain these results.

The significant leukopenia, neutropenia, and lymphopenia we observed in the zinc-deficient calves could be ascribed to the effect of zinc deficiency on the cell-mediated immunity as described by Ibrahim *et al*. [4] in sheep. This could be due to deterioration in the activity of serum thymulin, a thymic hormone required for maturation of T-helper cells [24]. Yousefichaijan *et al*. [25] further found that zinc was necessary for the development and activity of T-lymphocytes and its deficiency caused a reduction in cellular immunity, and Fraker *et al*. [26] found that zinc deficiency in mice was characterized by atrophy of lymphoid tissue that led to lymphopenia.

Zinc deficiency in young animals might be due to excess feeding of milk containing excess calcium that inhibits zinc absorption leading to secondary zinc deficiency, as reported by Sastry and Rao [27] and Radostits *et al*. [28].

Highly significant decreases in serum calcium levels and ALP activity were encountered in deficient calves, in accordance with those reported by Alsaad *et al*. [9]. In addition, Sharma and Joshi [20] demonstrated a significant correlation between serum calcium and ALP activity in zinc-deficient animals. Sastry and Rao [27] further found that calcium and phosphorous inhibited zinc absorption, leading to zinc deficiency. The downregulation of serum globulin observed in zinc-deficient calves could be attributed to mitogenesis dysfunction of B-prolymphocyte causing a decline in the serum concentration of immunoglobulins and the level of antibody response to T-dependent antigen [29].

Considering all these data, treatment of naturally zinc-deficient calves with oral administration of zinc oxide at the recommended dose returned the clinical and laboratory alterations of the diseased calves to normal.

## Conclusion

Natural zinc deficiency in calves caused clinical, hematological, and biochemical alterations, such as alopecia, skin abnormalities, macrocytic normochromic anemia, and an effect on cell-mediated immunity as indicated by leukopenia and lymphopenia. These alterations were improved by oral administration of zinc oxide.

## Authors’ Contributions

MME designed the experiment and reviewed the manuscript. AEM performed the experiment, collected samples, analyzed the data, and wrote the original draft. Both authors read and approved the final manuscript.
